# Quantitative Pupillometry for Intracranial Pressure (ICP) Monitoring in Traumatic Brain Injury: A Scoping Review

**DOI:** 10.1007/s12028-023-01927-7

**Published:** 2024-02-13

**Authors:** Karol Martínez-Palacios, Sebastián Vásquez-García, Olubunmi A. Fariyike, Chiara Robba, Andrés M. Rubiano, on behalf of the noninvasive intracranial pressure monitoring international consensus group

**Affiliations:** 1 Neuroscience Institute, Universidad El Bosque, Bogotá, Colombia; 2 Fundación para la Educación e Investigación Médica y Técnica en Emergencias “MEDITECH”, Cali, Colombia; 3 Universidad del Rosario, Bogotá, Colombia; 4 Stanford University School of Medicine, Palo Alto, CA USA; 5 Department of Anesthesia and Intensive Care, Policlinico San Martino, Genova, Italy

**Keywords:** Intracranial pressure (ICP), Monitoring, Traumatic brain injury (TBI), Intracranial hypertension, Noninvasive monitoring, Invasive monitoring, Quantitative pupillometry, Pupillometer

## Abstract

The neurological examination has remained key for the detection of worsening in neurocritical care patients, particularly after traumatic brain injury (TBI). New-onset, unreactive anisocoria frequently occurs in such situations, triggering aggressive diagnostic and therapeutic measures to address life-threatening elevations in intracranial pressure (ICP). As such, the field needs objective, unbiased, portable, and reliable methods for quickly assessing such pupillary changes. In this area, quantitative pupillometry (QP) proves promising, leveraging the analysis of different pupillary variables to indirectly estimate ICP. Thus, this scoping review seeks to describe the existing evidence for the use of QP in estimating ICP in adult patients with TBI as compared with invasive methods, which are considered the standard practice. This review was conducted in accordance with the Joanna Briggs Institute methodology for scoping reviews, with a main search of PubMed and EMBASE. The search was limited to studies of adult patients with TBI published in any language between 2012 and 2022. Eight studies were included for analysis, with the vast majority being prospective studies conducted in high-income countries. Among QP variables, serial rather than isolated measurements of neurologic pupillary index, constriction velocity, and maximal constriction velocity demonstrated the best correlation with invasive ICP measurement values, particularly in predicting refractory intracranial hypertension. Neurologic pupillary index and ICP also showed an inverse relationship when trends were simultaneously compared. As such, QP, when used repetitively, seems to be a promising tool for noninvasive ICP monitoring in patients with TBI, especially when used in conjunction with other clinical and neuromonitoring data.

**Supplementary Information:**

The online version contains supplementary material available at 10.1007/s12028-023-01927-7.

## Introduction

Neurological examination has been the cornerstone of detecting worsening conditions in neurocritical care patients, including traumatic brain injury (TBI) [1–3]. New-onset, unreactive anisocoria frequently occurs in these emergency situations and triggers a set of diagnostic (e.g., computerized tomography acquisition) and therapeutic (e.g., hyperosmolar therapy, hyperventilation, or decompressive craniectomy) measures to aggressively address a potentially life-threatening condition [1]. Intracranial pressure (ICP) monitoring is a current practice in the management of patients with TBI [3, 4], even though there is no evidence that invasive ICP monitoring has internal validity. In fact, its measurements are known to be highly variable within and between institutions, countries, and levels of training of the treating team. Intracranial hypertension (IH), due to sustained ICP elevations, is independently associated with increased morbidity and mortality [3]. A classical approach to the neurological trauma examination includes two parts: the Glasgow Coma Scale score and the pupil examination. Although the Glasgow Coma Scale score can be replicated with reasonable consistency, several studies have demonstrated that the same cannot be said for the manual evaluation of the patient’s pupillary light reflexes (PLRs), whose measurement may be biased by subjective estimations of pupillary size and reactivity, as well as other factors, such as different degrees of ambient light during the examination. This leads to wide measurement discrepancies, with only 33.3% of pupils scored as nonreactive by health care practitioners also being scored as nonreactive by quantitative pupillometry (QP) [5, 6]. As such, it would be useful to have an objective, unbiased, portable, and reliable tool for assessing PLR and the influence of ICP changes on their values.

Regarding invasive ICP monitoring strategies (via intraparenchymal or intraventricular routes) [7], their use is consistently mired by many factors, such as the unavailability of coagulopathy devices and the lack of experienced personnel in low-to-middle-income countries [8, 9]; the risk of catheter-related infections [10]; and the controversies surrounding appropriate timing of ICP monitoring, catheter placement, or catheter withdrawal [11]. To combat these problems, noninvasive modalities could be a reliable, cost-effective, and safe alternative in bedside monitoring [12]. Currently, there are many different methods for noninvasive ICP (nICP) estimation, including sonographic optic nerve sheath diameter (ONSD) measurement, transcranial Doppler (TCD)-derived indices [13], and the measurement of pupil size and other dynamic pupillary variables (e.g., neurologic pupillary index [NPi], latency, constriction velocity, and dilation velocity) [14]. Although the role of QP has been studied in the general intensive care unit population [15, 16], few works have demonstrated the relationship between ICP and changes in QP parameters, which may be of great importance given the frequency of IH in TBI, and its secondary consequences in terms of morbidity and mortality in those patients [4]. Specifically, this article seeks to characterize the evidence regarding QP’s ability to estimate nICP in the setting of TBI.

## Review Questions

The objective of this scoping review is to describe the extent and type of evidence regarding noninvasive methods for ICP monitoring in TBI using QP as compared with standard, invasive methods in the adult population. Applying the Population, Concept, and Context framework [17], the following specific questions were formulated:

1. Which methods are available for noninvasive ICP monitoring using QP?
2. What evidence exists for noninvasive ICP monitoring using QP versus invasive monitoring for ICP estimation?

## Methods

We decided to conduct a scoping review with the intention of exploring the depth of the literature, mapping and summarizing the evidence, identifying knowledge gaps and areas for future systematic reviews and other types of research, and assessing how the concept of QP in TBI has been studied in the scientific literature over time, given that the information available so far in this topic has been heterogeneous. This scoping review was conducted in accordance with the Joanna Briggs Institute methodology for scoping reviews [17]. This study did not involve human study participants research, and thus did not require institutional review board approval.

## Inclusion Criteria

### Participants

This scoping review considered studies including patients 18 years or older suffering from TBI, who underwent noninvasive ICP monitoring using QP and required diagnostic invasive ICP monitoring for intracranial pressure estimation. All studies in the pediatric population (less than 18 years) were excluded.

### Concept

The concept of this scoping review was to review studies that investigated nICP monitoring by QP in adult patients with all degrees of TBI (mild, moderate, and severe) as compared with the analysis derived from invasive methods. Topics in this concept include, but are not limited to, device features, methodological details, variables derived from said methods, the diagnostic accuracy of each method in detecting IH, the reliability of these methods, and the sensitivity and specificity of a specific QP method in the diagnosis of IH.

### Context

This scoping review did not consider the specific race, gender, or geographic location of participants in the selected studies. Given that the anatomy and pathophysiology of TBI within the pediatric population differ substantially from those of their adult counterparts, exclusion was determined solely by participant age, with only studies conducted in the adult population (18 years or older) being included.

### Types of Sources

The present scoping review assessed both experimental and quasi-experimental study designs including randomized controlled trials, nonrandomized controlled trials, before-and-after studies, and interrupted time-series studies. In addition, analytical observational studies including prospective and retrospective cohort studies, case–control studies, and analytical cross-sectional studies were considered for inclusion. This review also considered descriptive observational study designs including case series, individual case reports, and descriptive cross-sectional studies for inclusion. Qualitative studies that focus on qualitative data were also considered including, but not limited to, designs such as phenomenology, grounded theory, ethnography, qualitative description, action research, and feminist research. In addition, systematic reviews that met the inclusion criteria were also considered, depending on the research question.

### Search Strategy

An initial search in EMBASE and PubMed was undertaken, aimed at locating published studies in the adult population between January 2012 and June 2022 to obtain the most updated evidence and technological advances on the subject. Additionally, studies published in any language were included, as the available and useful literature is in a variety of languages. Studies that contained noninvasive monitoring with techniques other than QP were excluded. Studies containing invasive or noninvasive ICP monitoring for the diagnosis of IH from etiologies other than TBI were also excluded. A detailed search strategy from both databases is contained in Supplementary Appendix 1.

### Source of Evidence Screening/Selection

**Fig. 1:**
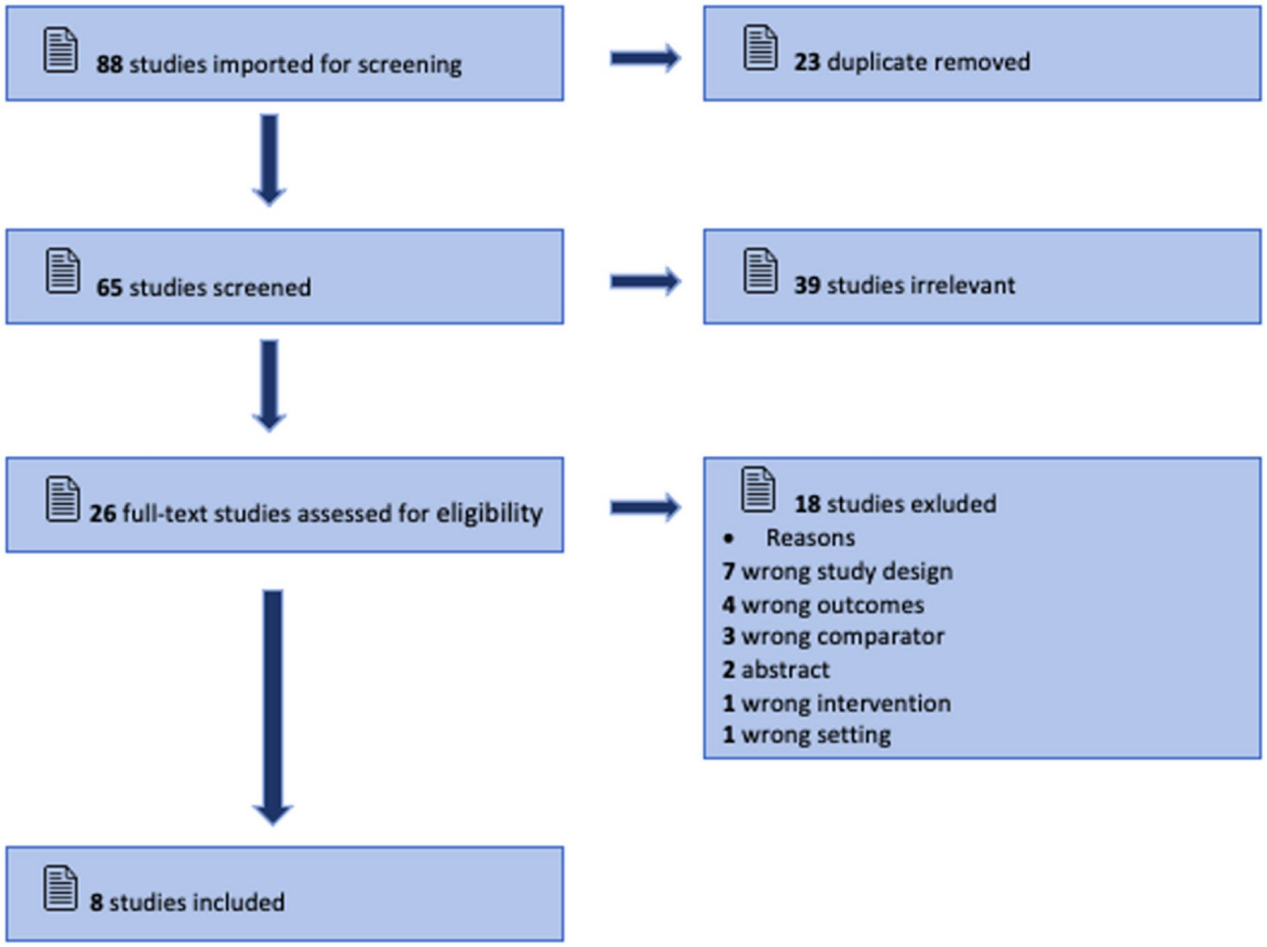
Extraction methodology

The initial EMBASE and PubMed search yielded 88 studies. All identified citations were collated and uploaded into Covidence, and 23 duplicated studies were removed. Studies were screened by two independent researchers (KM and OF) and one collaborator (SV). After examining 65 titles and abstracts for inclusion, 39 irrelevant studies were removed, 26 full-text studies were assessed for eligibility, and 18 studies were excluded for reasons described in Fig. 1. The results of the search are reported using the Preferred Reporting Items for Systematic Reviews and Meta-Analyses extension for Scoping Reviews checklist [18].

## Results

**Fig. 2:**
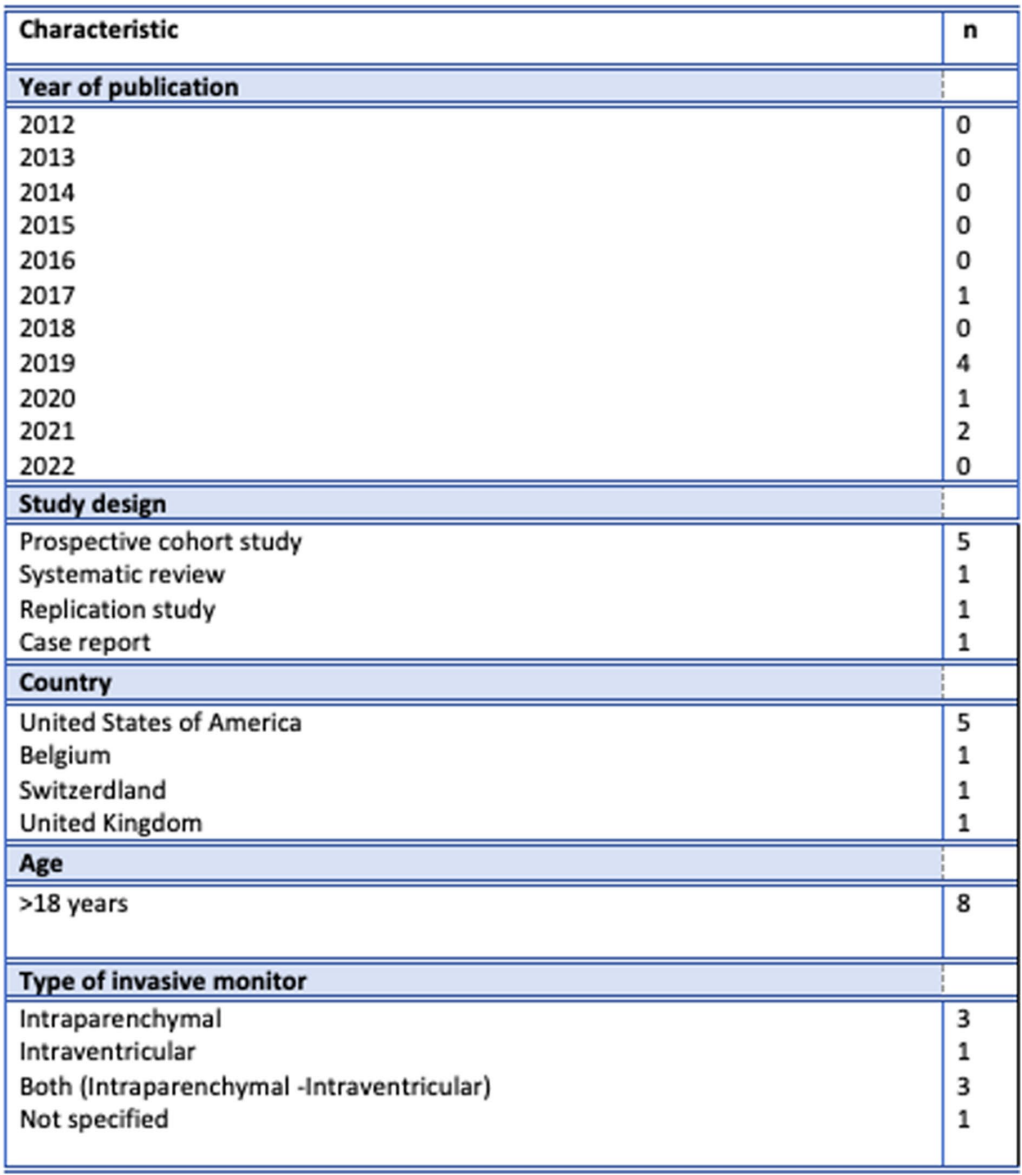
Characteristics of included publications

After reviewing and applying inclusion and exclusion criteria, eight studies were included for final analysis. Figure 2 provides the characteristics of the included publications. Table 1 provides the extracted information based on the formulated research questions.

**Table 1:** QP-based methods for ICP estimation and their correlation with invasive ICP

First author, year of publication	Country	Type of study	Number of participants	Technique/method of nICP estimation	Method of Iicp monitoring	nICP versus iICP monitoring	Conclusions
McNett M, 2017 [22]	United States	Cohort study	76 (43 TBI)	NeurOptics NPi-100 pupillometer provided an objective measure of CV, DV, PS, and latency, which are used to calculate Npi*	Intraparenchymal	Right and left Npi* were weakly but statistically significantly inversely correlated with iICP (*r* = − 0.126, * p* < 0.001 and * r* = − 0.225, * p* < 0.0001) Right Npi* and left CV were weakly but statistically significantly inversely correlated with iICP (*r* = − 0.195, * p* < 0.000 and * r* = − 0.199, * p* < 0.000, respectively) Right Npi* and left PS were weakly but statistically significantly positively correlated with iICP (*r* = 0.166, * p* < 0.000 and * r* = 0.133, * p* < 0.001, respectively) Right and left Npi* (*B* = 3.64, *t* = 5.96, * p* < 0.001; *B* = 3.40, *t* = 6.120, * p* < 0.001, respectively) were strongest predictors of iICP, with right and left CV also being significant predictors (*B* = 3.26, *t* = 4.17, * p* < 0.001; *B* = 2.04, *t* = 2.65, * p* < 0.005, respectively) Overall, the model was significant for predicting iICP, although rather weakly (*R*^2^ = 0.14, F(6) = 17.63, * p* < 0.001) Pupillometer values are significantly but weakly correlated with ICP (*r* = 0.13–0.23, * p* < 0.001)	PS was not a major predictor of iICP, contrary to numerous previous studies. However, in those studies, PLR was merely recorded as present or absent The addition of QP improves accuracy of examinations and correlates with iICP values, but correlations are weak overall
Robinson MB, 2021 [27]	United States	Case report	1	NeurOptics NPi-200 pupillometer provided an objective measure of CV, DV, PS, and latency to calculate NPi*	Intraparenchymal and Intraventricular	Patient iICP was > 20 mmHg, and, during this time, QP results showed slight asymmetry and decreased NPi* on the left. The left pupil became nonreactive between days 2 and 3	Abnormal NPi* in a comatose patient was found to be predictive of the need for surgical intervention NPi* has been associated with both increased iICP and subsequent spikes in iICP, supporting the concept that worsening NPi* may be a harbinger of herniation and, therefore, may be used to guide earlier intervention
Al-Obaidi, 2019 [24]	United States	Replication study/Case control study	273 (14 TBI)	NeurOptics NPi-200 pupillometer provided an objective measure of CV, DV, PS, and latency to calculate NPi*	Intraventricular	Using all paired iICP and pupillometer readings, elevated iICP (≥ 15 mmHg) was associated with significantly higher right (*p* = 0.03) and lower left (*p* < 0.0001) NPi, slower right (*p* < 0.0001) and left (*p* < 0.0001) CV, slower right (*p* < 0.0001) and left (*p* < 0.0001) DV, smaller right (*p* < 0.0001) and left (*p* < 0.0001) PS, and longer left (*p* < 0.0001) latency. Right latency showed no significant difference When using a mixed model to account for repeated measures, those with elevated iICP showed significantly higher NPi, smaller PS, longer latency, and slower CV and DV in both eyes	These results seem to suggest that elevated iICP may be manifest in the NPi* and CV, without significant changes in PS There is an inverse relationship between iICP values and NPi* (If the iICP value is greater or equal to 15 mm Hg), but PS is not affected by iICP values
Jahns FP, 2019 [20]	Switzerland	Cohort study	54 (sTBI)	NeurOptics NPi-200 pupillometer provided an objective measure of CV, DV, PS, and latency to calculate NPi*	Intraparenchymal	Average iICP increased (from 14 ± 5 mmHg at baseline to 30 ± 9 mmHg maximum) in the 6 h post-initiation of an IH episode and was associated with a clinically relevant decrease in NPi* (4.2 ± 0.05 at baseline to 2.8 ± 1.6 at minimum) Within the 15 observed episodes of osmotherapy, baseline iICP decreased from 29 ± 8 mmHg baseline to 12 ± 6 mmHg min and was associated with an increase in NPi* from 2.6 ± 1.7 at baseline to 4 ± 1.2 maximum Percentage of QP samples with abnormal NPi* value was higher in the refractory IH group (38%) than in the non-refractory IH group (1%) or normal iICP group (.5%) (*p* = 0.007) Only patients with IH showed no NPi* recovery, and all had poor overall recovery (GOS 1–3) at 6 months. In patients who had decompressive hemi-craniectomy, only those who showed NPi* recovery had good outcomes (GOS 4–5)	Abnormal NPi* is associated with increased severity of intracranial hypertension (and a worse 6-month outcome) The percentage of sampled NPi* values < 3 during ICP monitoring was significantly higher in patients with refractory IH (38%) than in those with non-refractory IH (1%) or no IH (0.5%) (*p* = 0.007) Differences in the NPi* values between the two eyes were frequent, with the lowest NPi* value being ipsilateral to focal injury in 62% of cases Sustained elevations of iICP > 20 mmHg are associated with a concomitant and clinically relevant decrease of quantitative NPi*, on average below normal values (NPi* < 3) The cumulative burden of abnormal NPi* was a marker of an increased severity of intracranial hypertension and a more complicated ICP course (requiring decompressive craniectomy)
Al-Mufti F, 2019 [23]	United States	Systematic review	100 studies (9 QP)	Varied	Intraventricular and Intraparenchymal	Abnormally high iICP (> 20 mm Hg) was correlated with a PLR that is reduced by 20% Abnormal PLR was reported to occur 15.9 h prior to the peak ICP	High ICP (> 20 mmHg) is correlated with a 20% decrease in pupil constriction Pupillometers are much more sensitive than manual scoring for small changes in PS Abnormal PLR noted 15.9 h ahead of peak ICP QP is not sufficient in isolation and must be combined with other non-invasive methods
Stevens AR, 2019 [25]	United Kingdom	Cohort study	40	NeurOptics NPi-100 pupillometer provided an objective measure of CV, DV, PS, and latency to calculate NPi*	Intraparenchymal	Significant events included iICP > 20 mmHg for 2 h or > 25 mmHg at any time, a change in NPi* > 1 point between two consecutive readings or a score < 3 at any time Right NPi* and left NPi* are moderately related to each other in event frequency (*r* = 0.48; * p* < 0.01), event duration (*r* = 0.64; * p* < 0.01) and strongly correlated in average event duration (*r* = 0.98; * p* < 0.01); however, both variables show weak, insignificant correlation to iICP for event frequency, duration, or average duration On average, there is a greater lag between NPi* and iICP events in the left eye (− 1 h; 95% CI [− 11, − 1]; * p* = 0.04) Of the 55 recorded iICP events, 20 had preceding NPi* events in both eyes. 33 had a prior NPi* event in at least 1 eye and 22 had no prior NPi* event in either eye There is a weak, insignificant relationship between changes in NPi* and iICP (O* r* = 3.6 95% CI [.93, 13.53]; * p* = 0.07)	There is a weak, statistically insignificant inverse relationship between changes in NPi* and iICP
Robba C, 2020 [26]	Belgium	Cohort study	100 (30 TBI)	NeurOptics NPi-200 pupillometer provided an objective measure of CV, DV, PS, and latency to calculate NPi*	Intraparenchymal and Intraventricular	There was no correlation between iICP and NPi* (*r* = − 0.0.16; * p* = 0.37). The AUC for NPi* in estimating IH was 0.61 [95% CI 0.49–0.83] (which was much lower than that of ONSD (0.78), PI (0.79), or eICP (0.83)) NPi* < 4.0 had 61% sensitivity and 73% specificity in predicting IH Only 4 (13.3%) of the 30 patients showed abnormal NPi* values even though 15 (50%) had IH (≥ 20 mmHg) according to EVD measurements In the overall cohort, ONSD had a significant but weak correlation with NPi* (*r* = -0.22;* p* = 0.02). PI had a significant, weak-to-moderate correlation with NPi* (*r* = -0.27; * p* = 0.006), and eICP had a significant, weak-to-moderate correlation with NPi* (*r* = − 0.29; * p* = 0.003)	This study demonstrated that a relationship exists between NPi* values < 3 and increased iICP, with abnormal NPi* values present before the increase in iICP NPi* had the lowest accuracy for estimating intracranial hypertension, and its addition to the other indices did not significantly improve their accuracy NPi* had a weak correlation in TBI (but good in other diseases, i.e. SAH)
Singer K, 2021 [21]	United States	Cohort study	135 patients (sTBI: 36; mTBI: 30)	An unspecified NeurOptics pupillometer provided an objective measure of CV, DV, PS, and latency to calculate NPi*	Not specified	Changes in pupillary dynamic values proved more effective in differentiating sTBI, mTBI, and nTBI than absolute PS NPi* (sTBI vs. mTBI and nTBI) was significant bilaterally on day 1 of hospital stay (*p* < 0.01 except right nTBI, * p* < 0.05) % change in PS (sTBI vs. mTBI and nTBI) was significant bilaterally on the day 1, 2, and 3 of hospital stay (*p* < 0.001) CV (sTBI vs. mTBI and nTBI) was significant bilaterally on the day 1, 2, and 3 of hospital stay (*p* < 0.001) Mean CV (sTBI vs. mTBI and no TBI) was significant bilaterally on the day 1, 2, and 3 of hospital stay (*p* < 0.001) DV (sTBI vs. mTBI and nTBI) was significant bilaterally only on day 2 of hospital stay (*p* < 0.001)	An increase in iICP may be associated with decreased PLR Pupillometry may be more effective at estimating iICP in cases of elevated ICP Dynamic measurements of pupillometry reliably differentiated sTBI from mTBI on post-injury days 2 and 3. Interestingly, however, these same measurements did not correlate to iICP in patients with sTBI

*AUC* area under curve, *CI* confidence interval, *CN* cranial nerve, *CV* pupil constriction velocity in response to light (in mm/s), *DV* pupil dilation velocity in absence of light (in mm/s), *eICP* non-invasively estimated ICP, *EVD* extraventricular drain, *GOS* Glasgow Outcome Scale (non-extended), *IH* intracranial hypertension, *ICP* intracranial pressure, *iICP* invasive ICP, *MCV* maximum constriction velocity (in mm/s), *mTBI* mild traumatic brain injury, *nICP* non-invasive intracranial pressure, *NPi* neurological pupillary index, *nTBI* no TBI, *ONSD* optic nerve sheath diameter, *OR* odds ratio, *PLR* pupillary light reactivity/response, *PS* pupil size (in mm), *QP* quantitative pupillometry, *ROC* receiver operating characteristic curve, *SAH* subarachnoid hemorrhage, *sTBI* severe traumatic brain injury

*NPi is derived using an algorithm that takes quantitative pupillometer values obtained from TBI patients and compares them to the mean distribution of those obtained from healthy subjects. Values are standardized into Z-scores and combined to create an NPi* score that ranges from 0 to 5. An NPi* score greater than 3 indicates normal pupillary reactivity, whereas a score less than 3 reflects an abnormal pupillary light reflex, which can be associated with direct damage to CN III or indirect brain damage

### Noninvasive ICP Monitoring Using QP-Derived Parameters

There was profound homogeneity among the analyzed studies in the device selected for pupillometry, with all studies describing the use of the NeurOptics brand device, a noninvasive, handheld optical scanner that provides reliable and objective measurements of pupillary size (PS), symmetry, and reactivity [19]. This device uses an infrared camera that fixes a calibrated light stimulus of fixed intensity (1000 lx) and duration (3.2 s) on the pupil as the infrared camera captures 90 images, allowing for a rapid and precise (within 0.05 mm) measurement of pupil size and a series of dynamic pupillary variables, including the pupil’s maximum and minimum size (minimum right and minimum left), percent constriction (percent right and percent left), constriction velocity (CV) (CV right and CV left), maximum constriction velocity (MCV) (MCV right and MCV left), dilation velocity (DV) (DV right and DV left), and latency for the right and left pupils, respectively [20, 21]. The device records serial numeric readings of both eyes, allowing for the visualization of trends and subtle changes in pupillary responses over time [22]. This noninvasive tool also tabulates the NPi, a proprietary index created by an algorithm that incorporates a number of the previously described variables, combining and comparing them against a mean derived from a reference distribution of healthy study participants. Values are standardized to fall within a scale set from zero to five. An NPi value greater than three indicates normal pupillary reactivity, and a value less than three suggests abnormal PLRs. By the same logic, a value of zero indicates a nonreactive, immeasurable, or atypical response [19, 22]. To use the device, the examiner holds it in front of the patient’s eye, lifting up the eyelid as needed to visualize the pupil. The device, using the light and infrared camera as explained above, and then automatically assigns both pupils an NPi value using the aforementioned quantitative metrics [21].

The NPi index was quantified in each of the studies reviewed and was the main comparator against invasive ICP for the estimation of intracranial pressure (Table 1). However, in four of the studies, other variables were considered. McNett et al. [22] included variables such as CV and pupil size in their analysis, and Al-Mufti et al. [23] considered variables such as pupil size and CV in percentage terms. The study by Al-Obaidi et al. [24] considered CV, DV, PS, and latency, being the only study to consider the last variable. In a series of studies, the NPi was used as the only comparator for pupillometry against invasive ICP (iICP) [20, 25–27]. Interestingly, Singer et al. [21] was the only study that determined the maximum and minimum pupil size as well as the MCV. No other study that met the inclusion criteria made such a distinction when using variables other than NPi.

### nICP by QP Versus iICP for ICP Estimation

A prospective cohort study in 76 patients (43 TBI) using NPi, PS, and CV found that right and left NPi had an inversely proportional relationship to ICP that was weakly but statistically significant (*r* =  − 0.126, *p* < 0.001 and *r* =  − 0.225, *p* < 0.000, respectively). Right NPi and left CV were also weakly but statistically significantly inversely correlated with ICP (*r* =  − 0.195, *p* < 0.000 and *r* =  − 0.199, *p* < 0.000, respectively). Right NPi and left PS were weakly but statistically significantly positively correlated with ICP (*r* = 0.166, *p* < 0.000 and *r* = 0.133, *p* < 0.001, respectively). Right and left NPi values (*p* < 0.001 for both) were the strongest predictors, with right and left CV also being significant predictors (*p* < 0.001 and *p* < 0.005, respectively). High NPi and CV values correlated with normal iICP, and there was no significant correlation with ICP for PS, particularly when compared to correlation values for NPi and CV. Pupillometer values as a whole were significantly but weakly correlated with ICP (*r* = 0.13–0.23, *p* < 0.001) (Table 1); however, the results were not adjusted for the multiple measurements made [22].

A replication study with a case–control design in 273 patients (14 TBI) utilized NPi, CV, DV, PS, and latency and defined normal ICP as less than 15 mm Hg and elevated ICP as greater than or equal to 15 mm Hg. They compared QP to invasive ICP monitoring using an intraventricular catheter. This study found that, in the right eye, an ICP of less than 15 mm Hg was significantly associated with lower NPi (*p* = 0.03), faster CV (*p* < 0.0001), faster pupil dilation as measured by DV (*p* < 0.0001), and larger pupil size (*p* < 0.0001). In the left eye, ICP less than 15 mm Hg was significantly associated with higher NPi (*p* < 0.0001), faster CV (*p* < 0.0001), faster DV (*p* < 0.0001), and larger pupil size (*p* < 0.0001). By contrast, in the right eye, an ICP greater than or equal to 15 mm Hg was significantly associated with lower NPi (*p* = 0.0300), slower CV (*p* < 0.0001), and slower DV (*p* < 0.0001). Similarly, in the left eye, an ICP greater or equal to 15 mm Hg was significantly associated with lower NPi (*p* < 0.0001), CV, and DV (both *p* < 0.0001). When using a mixed model to account for repeated measures, those with elevated ICP showed significantly higher NPi, smaller pupil size, longer latency, and slower constriction and dilation velocities in both eyes. It is worth noting that the directional correlation between elevated ICP and NPi values was inconsistent according to this study's results, but that, overall, lower NPi values were associated with increased ICP [24].

In a prospective cohort study with 54 patients with TBI, an episode of elevated ICP was defined as ICP greater than 20 mm Hg for more than 10 min, and IH was defined as nonrefractory (responsive to medical management, including osmotherapy, with ICP returning to less than 20 mm Hg) or refractory (persistent, sustained ICP elevation greater than 25 mm Hg and requiring surgical decompression). First, these authors calculated the total cumulative burden of abnormal (< 3) NPi values at the three different timepoints before a patient's peak ICP. The NPi values used for analysis were those of the lowest NPi value between both eyes, and, in cases where the NPi was abnormal on one side but normal on the other, the lowest value overall was considered (Table 1). ICP increases were associated with concomitant and clinically relevant decreases in NPi from a baseline of 4.2 ± 0.5 to 4.0 ± 0.6 at T1 (*p* = 0.14), 3.5 ± 1.2 at T2 (*p* < 0.0001), and 2.8 ± 1.6 at minimum NPi (*p* < 0.0001). Differences in the NPi values between the two eyes were frequent, with the lowest NPi value being ipsilateral to the focal injury in 62% of cases. Within the 15 observed interventions with osmotherapy, baseline ICP (at the beginning of osmotherapy) decreased from 29 ± 8 to 12 ± 6 mm Hg (ICP minimum; *p* < 0.0001), which was associated with a concomitant increase in baseline NPi from 2.6 ± 1.7 to 3.1 ± 1.5 (T1; *p* = 0.07), 3.7 ± 1.3 (T2; *p* = 0.0006), and 4 ± 1.2 (NPi maximum; *p* < 0.0001). Interestingly, the percentage of QP samples with abnormal (< 3) NPi values was higher in the refractory IH group (38%) than in the nonrefractory IH group (1%) or normal ICP group (5%) (*p* = 0.007) [20].

In a systematic review of multimodality monitoring in neurocritical care, abnormally high ICP (> 20 mm Hg) was correlated with a pupil response that is reduced by 20% of normal constriction, with abnormal pupillary activity reported as occurring 15.9 h prior to ICP peak, on average [23].

A prospective study including 40 patients with TBI aimed to compare NPi and its relationship to iICP as measured using an intraparenchymal device (Table 1), with hourly pupillometry and ICP readings over a period of 72 h. Significant events included ICP > 20 mm Hg for 2 h, ICP > 25 mm Hg at any time, a change in NPi > 1 point between two consecutive readings, or an NPi value < 3 at any time. Of the 55 recorded ICP-related events, 26 had a corresponding prior NPi event in the left eye and 27 had a corresponding prior NPi event in the right eye. There were 20 ICP events that had corresponding prior NPi events in both eyes, and 33 had a corresponding prior NPi event in at least one eye. On average, there was a greater lag in the left eye (based on 26 occasions in which an ICP event was preceded by an NPi event) than in the right eye (based on 27 occasions (mean difference − 1 h; 95% confidence interval [CI] [− 11 to − 1]; *p* = 0.04]). These results demonstrate a weak and statistically insignificant relationship between changes in NPi and ICP (odds ratio 3.36, 95% CI [0.93–13.53] *p* = 0.07). Additionally, this study finds that the length of lag in both eyes is right-skewed, with a median of six hours in the right eye and 12.5 h in the left eye (Table 1) [25].

Another cohort study assessed multimodal, noninvasive modalities for measuring IH in 100 patients (30 TBI), comparing TCD (through pulsatility index, [PI] and estimated ICP), ONSD (via ultrasound), and QP (through NPi). IH was defined as ICP > 20 mm Hg, and the external ventricular drain was closed during ICP measurements to ensure the accuracy of invasive ICP (iICP) measurements. In the TBI subgroup, the area under the curve (AUC) for NPi in estimating IH was.61 [95% CI 0.49–0.83], which was much lower than that of ONSD (0.78), PI (0.79), or estimated ICP (0.83). An NPi value of < 4.0 exhibited a 61% sensitivity and 73% specificity in predicting IH, the lowest combination of sensitivity and specificity of the four aforementioned parameters in the comparison. Uniquely, the highest AUC for the entire cohort (AUC = 0.91) and for just patients with TBI (AUC = 0.92) was achieved with a combination of ONSD and estimated ICP, which was not improved by the addition of NPi nor PI nor both together. In the overall cohort, ONSD had a significant but weak correlation with NPi (*r* =  − 0.22; *p* = 0.02), and PI and estimated ICP had a significant, weak-to-moderate correlations with NPi (*r* =  − 0.27; *p* = 0.006 and *r* =  − 0.29; *p* = 0.003, respectively) (Table 1) [26].

A prospective cohort study analyzed the efficacy of noninvasive technologies in triaging patients with TBI and estimating ICP in 135 patients (66 TBI) classified as having a severe TBI (sTBI, 36), mild TBI (mTBI, 30), or no TBI (nTBI, 66). In this study, they did not specify the method of invasive ICP monitoring. NPi (sTBI vs. mTBI and nTBI) was significantly higher in the sTBI group bilaterally on day one of patient hospital stay (*p* < 0.01 except right eye in the nTBI group *p* < 0.05). Also, the percent change in pupil diameter, CV, and mean CV were significantly lower in the sTBI group bilaterally on the first three days of patient hospital stay (all *p* < 0.001). DV (sTBI vs. mTBI and nTBI) was only significantly lower bilaterally on day two of patient hospital stay (*p* < 0.001). Thus, dynamic measurements of pupillometry reliably differentiated severe TBI from more mild brain injuries on postinjury days two and three; however, these same measurements did not correlate to ICP in patients with severe TBI. In fact, many patients with sTBI had lower ICPs than their counterparts with less severe injuries. Further, changes in pupilar dynamic values proved more effective in differentiating sTBI than absolute pupil size [21].

Finally, a case report study analyzed decision-making for decompressive craniectomy in a patient with a TBI aided by multimodality monitoring (TCD and QP). The patient’s ICP was > 20 mm Hg during his first three days of admission, and the QP results showed slight asymmetry and decreased reactivity as measured by left NPi on day one, progressing to nonreactivity on days two and three. PbO2 began trending toward 1 on day three as well. These worsening parameters led to the decision to pursue a left-sided decompressive craniotomy (DC). After DC, his ICP remained below 10 mmHg, PbO2 increased, and left NPi and pupil size returned to normal within hours [27].

## Discussion

Serial QP parameters, specifically NPi, CV, and MCV, may be key to tracking the development of elevated ICP or IH and could be correlated with the severity of IH (e.g., refractory IH). Nevertheless, that predictive ability seems to be slightly better for monitoring established, nonrefractory IH, given a weak-to-moderate correlation between QP and absolute ICP values.

Examining pupillary light reactivity has been one of the main clinical tools for assessing deterioration risk in TBI for decision-making at bedside. In fact, aggressive management strategies (both medical and surgical) and prognostic tools with validated scores such as Corticosteroid Randomization after Significant Head Injury and IMPACT (International Mission of Prognosis and Analysis of Clinical Trials) include pupillary response in their analyses [28]. A detailed description of pupillary physiology and anatomical features is beyond the scope of this article; however, it is important to consider a few key points in order to establish the role of the PLR in TBI. Perhaps the most well-known scenario is that of a lateral descending transtentorial herniation, in which anisocoria develops due to the close anatomical relationship between the medial temporal lobe and cranial nerve III (CN III) [29]. Notwithstanding, disturbances in other structures involved in the whole PLR circuitry play a role in the pathophysiology of patients with TBI, as well, even without the occurrence of a life-threatening process such as cerebral herniation. Among these, intracranial relay and regulatory structures such as the suprachiasmatic hypothalamic nucleus, paraventricular nucleus, dorsomedial hypothalamus, periaqueductal gray matter, pretectal area, dorsal raphe nucleus, locus ceruleus, ciliary ganglion, and efferent pathways other than CN III such as the Edinger-Westphal and accessory nuclei play crucial roles in both sympathetic and parasympathetic pupillary responses [30]. All of these structures are prone to damage by elevated ICP and IH, and thus, QP may represent an objective way to indirectly assess the effects of ICP crisis on this complex functional unit.

As previously stated, all reviewed studies employed brand-licensed automated pupillometers (NeurOptics-100 and NeurOptics-200), which register different variables, all of which are related to the PLR, to calculate the NPi [19, 20]. Of these values, the NPi is the most influenced by ICP changes, followed by the CV and MCV. As such, currently and to the best of our knowledge, these indices seem to be the most reliable methods for nICP analysis using QP exclusively. However, monitoring these parameters’ trends, particularly NPi, by multiple and serial measurements rather than in isolation may allow prediction of patient worsening, and, in this specific setting, prediction of elevated ICP/IH even from 12 to 16 h before detection by invasive transducers [25, 27]. In addition, the findings from Jahns et al. showed that monitoring NPi trends and the cumulative burden of abnormal NPi could serve as markers of increased severity of IH and of a more complicated ICP course (requiring surgical management strategies like decompressive craniectomy) [20].

Although it was not one of the main objectives of this scoping review, the role of the NPi as a prognostic tool found in the aforementioned study deserves special mention as only patients with IH showed no NPi recovery during hospitalization, and all had poor overall recovery (GOS 1–3) at six months post-discharge. In patients who had a decompressive hemi-craniectomy, only those who showed NPi recovery during hospitalization had good outcomes (GOS 4–5) (Table 1) [20]. In-depth considerations regarding the use of QP for prognosis estimation in TBI and other diseases can be reviewed elsewhere [31, 32].

According to the analyzed evidence, NPi was found to have an inverse relationship with ICP values in all studies, with decreasing NPi correlated, although not consistently, with increasing ICP, particularly when measured as a trend. A big proportion of studies exhibited NPi less than three in cases of elevated ICP (defined consistently as ICP > 20 mm Hg). However, two studies found correlations for different NPi and ICP values: one found an inverse relationship between NPi and ICP for ICP values greater than or equal to 15 mmHg, and the other showed that moderate sensitivity and specificity for IH was obtained for NPi < 4 [24, 26]. These findings need to be considered in future studies, especially in light of the NPi values described in Robba et al., stating the enormous importance of having a higher threshold for “abnormal NPi”’ in earlier diagnostic (brain imaging) and therapeutic measures. Nevertheless, serial trend assessment is more important than an individual value in any given time period [20, 25, 26, 33].

QP may also have a role in differentiating TBI severity (mild vs. severe), which could translate into the presence or absence of intracranial injury (e.g., epidural or subdural hematoma). In this setting, this could be a method for establishing risk-based classification of TBI via the effects of ICP on PLR circuitry [34]. Given that NPi can be assessed individually in each eye and that differences in ICP values between both eyes were frequently reported, QP could also be useful for perceiving focal ICP changes on the side of the injury, and thus, screening of elevated ICP/IH in cases of suspected intracranial compartment syndrome (Table 1). In addition, the same study by Jahns et al. demonstrated a dynamic response of the NPi to ICP hyperosmolar therapy, with treatment leading to a normalization of NPi values [20]. This constitutes an additional role for QP at the bedside in monitoring the response to management strategies in patients with TBI with corresponding IH.

By contrast, QP has some potential and important confounders that need to be considered in its use for patients with TBI [35]. Sedative medications and analgesics, particularly opioids, induce changes in some QP variables that can be mistakenly attributed to ICP increases or decreases. It is worth mentioning that the study by Jahns et al. was the only study in this review that accounted for these confounders, describing that NPi appears to be less affected by those medications in comparison to other QP-derived variables such as pupil size and percentage constriction. They go on to note that the infusion dose of propofol was kept under 4 mg/kg in their study, reducing the probability of drug-induced PLR changes [20, 36]. Other confounders include cranial nerve diseases, which are prevalent in diabetes mellitus and raise questions about whether NPi is less reliable for ICP screening in patients with diabetes. Hypoxemia, hypercarbia, differences in circadian rhythms, ambient light, and pain, among others, need to also be considered in future trials designed to assess QP for nICP estimation [35]. Moreover, as pointed out by Robba et al., QP accuracy may vary depending on a particular disease due to the heterogeneity of pathophysiological processes in each condition. NPi had a weak correlation with ICP in TBI that was stronger in patients with aneurysmal subarachnoid hemorrhage (aSAH). This situation may be explained, in the authors’ opinion, by different disease-specific contributions to the increased ICP (mainly hydrocephalus in SAH vs. intracranial hemorrhage or brain edema in TBI). This can be ameliorated by using multiple nICP techniques that track each of these processes instead of using QP in isolation [26].

Finally, we would like to point out that, by the time of this article’s revision process, the Outcome Prognostication of Acute Brain Injury using the Neurological Pupil Index (ORANGE) study [37] about NPi for outcome prognostication in people with acute brain injury was published. Although the main endpoint was functional neurological outcome and mortality (which was outside of the aim and objectives of our work), and no iICP and QP specific correlations were given specifically for patients with TBI, we want to stress the importance of this study for the neurocritical care field, and we invite the scientific community to perform more research work on outcome determinants in TBI populations identified from pupillary metrics that are not only correlated with ICP changes, but may be also correlated with pupillary reactivity pathway structural injuries, seizures, among other factors.

## Limitations

Our review has several limitations. First, only studies in patients 18 years or older were included, potentially leaving out valuable information from patients 16 years and older who are also considered part of the “adult population.” Secondly, as a matter of a scoping review design, in-depth statistical analyses or risk-of-bias assessments that are usually performed in systematic reviews were not done in our study. This may represent a weakness for data interpretation in terms of diagnostic accuracy and nICP-iICP correlation comparison between studies. Third, narrative reviews, which may contain expert opinions and valuable information from other sources, were not included in our search. Finally, we only included studies in patients with TBI. As such, the analyses and conclusions derived from this work cannot be extrapolated to other neurocritical care patient populations (e.g., aSAH, ischemic stroke, or intracerebral hemorrhage).

## Conclusions

Quantitative pupillometry–derived parameters, specifically NPi, followed by CV and MCV, seem to have a potential role in IH prediction and grading of IH severity when analyzed serially, with NPi specifically having an inverse relationship with ICP. As such, QP, when used repetitively, seems to be a promising tool for nICP monitoring in patients with TBI, especially in conjunction with other clinical and neuromonitoring data. Even so, the field needs further studies that consider the analysis of different NPi values and their correlation with ICP, confirm the efficacy of QP in IH prediction, and assess the effects of ICP on other physiological parameters (e.g., cerebral perfusion pressure, cerebral oxygenation).

### Supplementary Information

Below is the link to the electronic supplementary material.

Supplementary file1 (DOCX 128 kb)
