# Supplementary figures and images for: Quantitative Pupillometry for Intracranial Pressure (ICP) Monitoring in Traumatic Brain Injury: A Scoping Review
Source: Neurocrit Care. 2024 Feb 13;41(1):255–71. doi: 10.1007/s12028-023-01927-7 (PMC11335905; doi:10.1007/s12028-023-01927-7)

**Appendix 1. EMBASE search strategy**

**
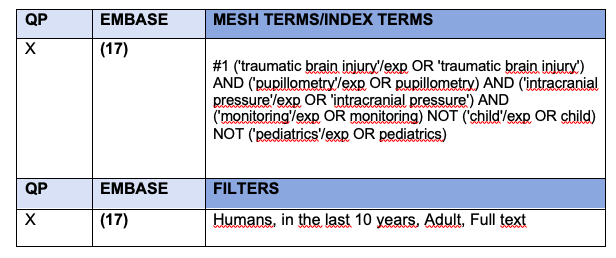
**

**Appendix 2. PUBMED search strategy**

**
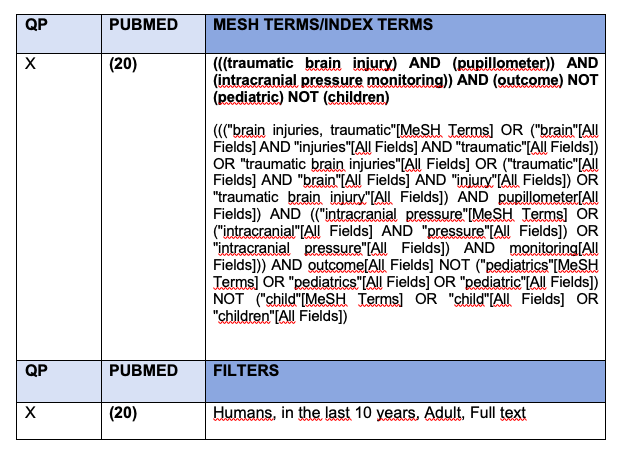
**

Supplement: Supplementary file 1 — Supplementary file1 (DOCX 128 kb) [file 12028_2023_1927_MOESM1_ESM.docx]
